# Leaving the Excision Site Open After Complete Excision of a Large Paracervical Leiomyoma With Fumarate Hydratase (FH)-Deficient Morphology May Decrease the Risk of Developing Renal Cell Carcinoma

**DOI:** 10.7759/cureus.101803

**Published:** 2026-01-18

**Authors:** Daniel Leung, Hamid Sanjaghsaz, Rehan Subramanyam

**Affiliations:** 1 Women's Health, Kentucky College of Osteopathic Medicine, Pikeville, USA; 2 Obstetrics and Gynecology, Michigan State University, East Lansing, USA; 3 Obstetrics and Gynecology, Touro College of Osteopathic Medicine, Great Falls, USA

**Keywords:** abdominopelvic pain, fumarate hydratase (fh) gene mutation, leiomyoma with bizarre nuclei, renal cell carcinoma (rcc), viscero-sympathetic convergence

## Abstract

Hereditary leiomyomatosis and renal cell cancer (HLRCC) syndrome is a rare autosomal dominant disorder characterized by cutaneous leiomyomas, uterine leiomyomas, and an increased risk of renal cell carcinoma. HLRCC may be preliminarily suspected through characteristic histopathologic features, but definitive diagnosis requires germline fumarate hydratase (FH) mutation testing. We report the case of a 41-year-old African American carrier of HLRCC phenotype who was diagnosed following hysterectomy and pathologic evaluation of a large leiomyoma in an unusually low-lying location of the uterus, demonstrating FH-deficient morphology. This case highlights potential links to the pathophysiology of how HLRCC-associated renal cell cancers may arise from FH-deficient leiomyomas around a decade later. Leaving the excision site open after complete excision of this leiomyoma with FH-deficient morphology may decrease the risk of developing renal cell carcinoma.

## Introduction

Hereditary leiomyomatosis and renal cell cancer (HLRCC) syndrome is a very rare genetic syndrome with an estimated prevalence of one in 200,000 [1]. Currently, an estimate is that HLRCC affects approximately 300 families globally [2]. HLRCC results from heterozygous germline mutations in fumarate hydratase (FH), an enzyme that converts fumarate to malate in the Krebs cycle [3]. Autosomal dominant mutations, mosaicism, or sporadic gene alterations predispose affected individuals to develop cutaneous leiomyomas, uterine leiomyomas, and renal cell carcinoma (RCC) with intracellular fumarate accumulation contributing to tumorigenesis [4]. RCC, most commonly of type 2 papillary histology, represents the most aggressive manifestation of HLRCC and occurs in approximately 10-16% of affected patients [5]. The histopathology of FH-deficient leiomyoma includes prominent eosinophilic nucleoli with perinucleolar halos, which may be due to the dysregulation of the biochemical pathway regulated by FH and represents signs of epigenetic changes. The FH-deficient morphology indicates that there is a predisposition to an aggressive form of renal cell cancer that requires early intervention, like surgery and genetic counseling. The median age at diagnosis of HLRCC-associated RCC is 44 years. Up to 85% of women with HLRCC develop larger and more numerous uterine leiomyomas at a younger age when compared to women in the general population. The median age at diagnosis of HLRCC syndrome is 30 years of age. The percentage of women who were carriers of the mutation causing HLRCC syndrome and had a myomectomy or hysterectomy by 40 years of age or younger was as high as 70% [6]. This case’s unique contribution is that it provides new insight into the possible link between the development of FH-deficient leiomyoma and RCC, given the location of this paracervical FH-deficient leiomyoma, its large size, and its proximity to the urinary system. A unique intraoperative decision was made to leave the orifice open after removal of the leiomyoma, which may provide a better prognostic outcome.

## Case presentation

We describe the case of a 41-year-old African American female who presented to the obstetrics and gynecology department with abdominopelvic pain and dysuria. Vital signs were within normal limits. A physical exam was performed, including a vaginal exam. No skin lesions were noted on exam. The OB/GYN was able to palpate some abnormality on a vaginal exam, but could not fully appreciate the mass that was palpated, and an ultrasound was ordered. She subsequently presented to the Emergency Department with right lower quadrant abdominal pain and right flank pain. She reported an acute onset of pain two days prior with progressive worsening. At presentation, the pain was rated 7/10 and described as sharp pressure in the right lower quadrant, associated with nausea and a decreased appetite. She denied diarrhea, constipation, hematuria, and hematochezia. She had no prior abdominal surgeries. She reported a history of uterine leiomyomas under evaluation, without prior episodes of comparable pain. She had no vaginal rashes or vaginal bleeding. Abnormal vaginal discharge was reported. The patient had no significant family history. The initial differential diagnosis for abdominopelvic pain, dysuria, RLQ abdominal pain, and right flank pain is broad and can include appendicitis, PID, ectopic pregnancy, endometriosis, leiomyoma, diverticulitis, and pyelonephritis.” Once the atypical uterine leiomyoma was visualized by ultrasound, and surgery was undertaken with pathological specimens examined showing the bizarre nuclei, there was a strong suspicion of HLRCC. Germline FH mutation testing has not yet performed.

**Figure 1:**
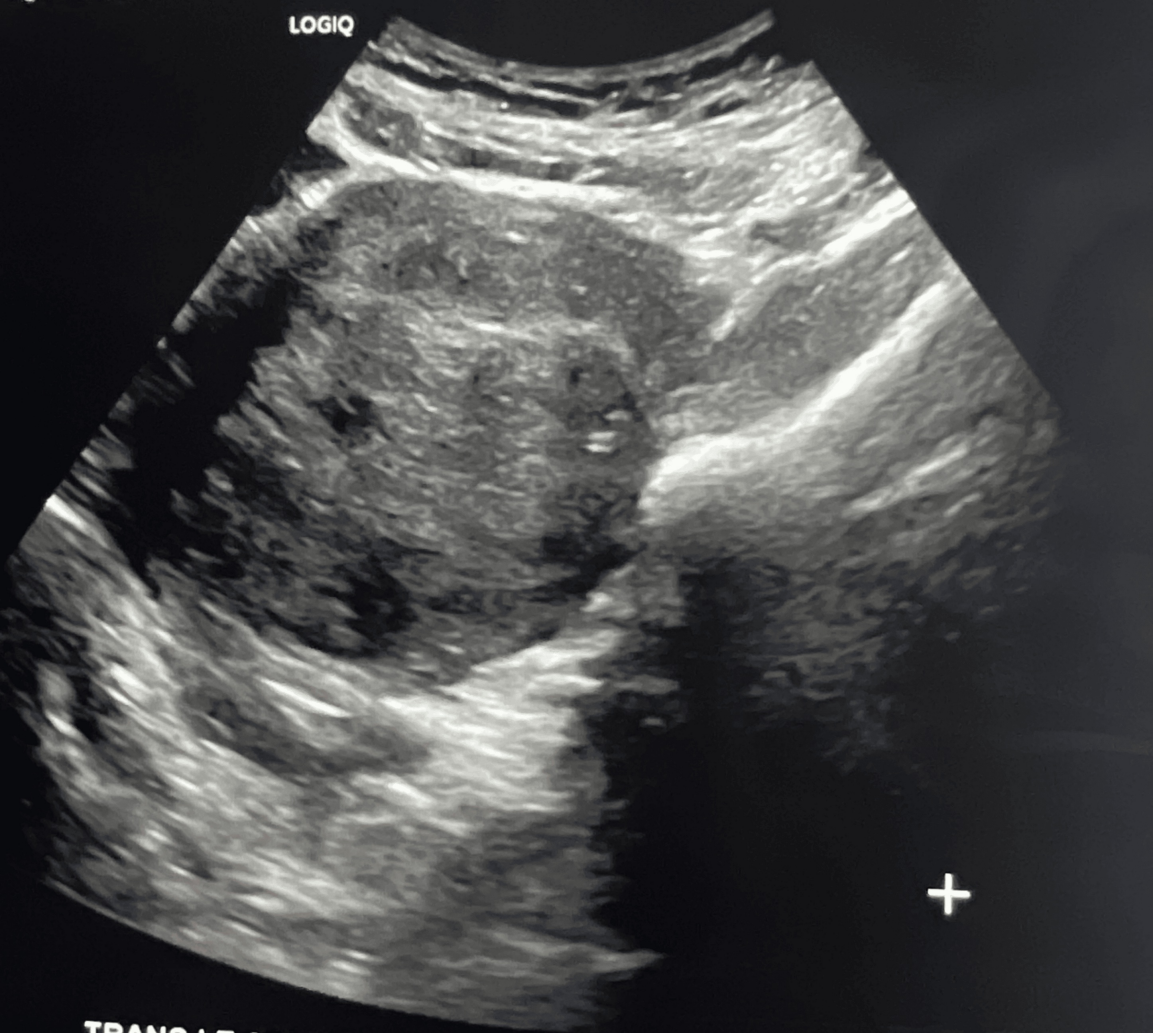
Ultrasound showing fumarate hydratase (FH)-deficient leiomyoma in the lower uterine section (paracervical)

**Figure 2:**
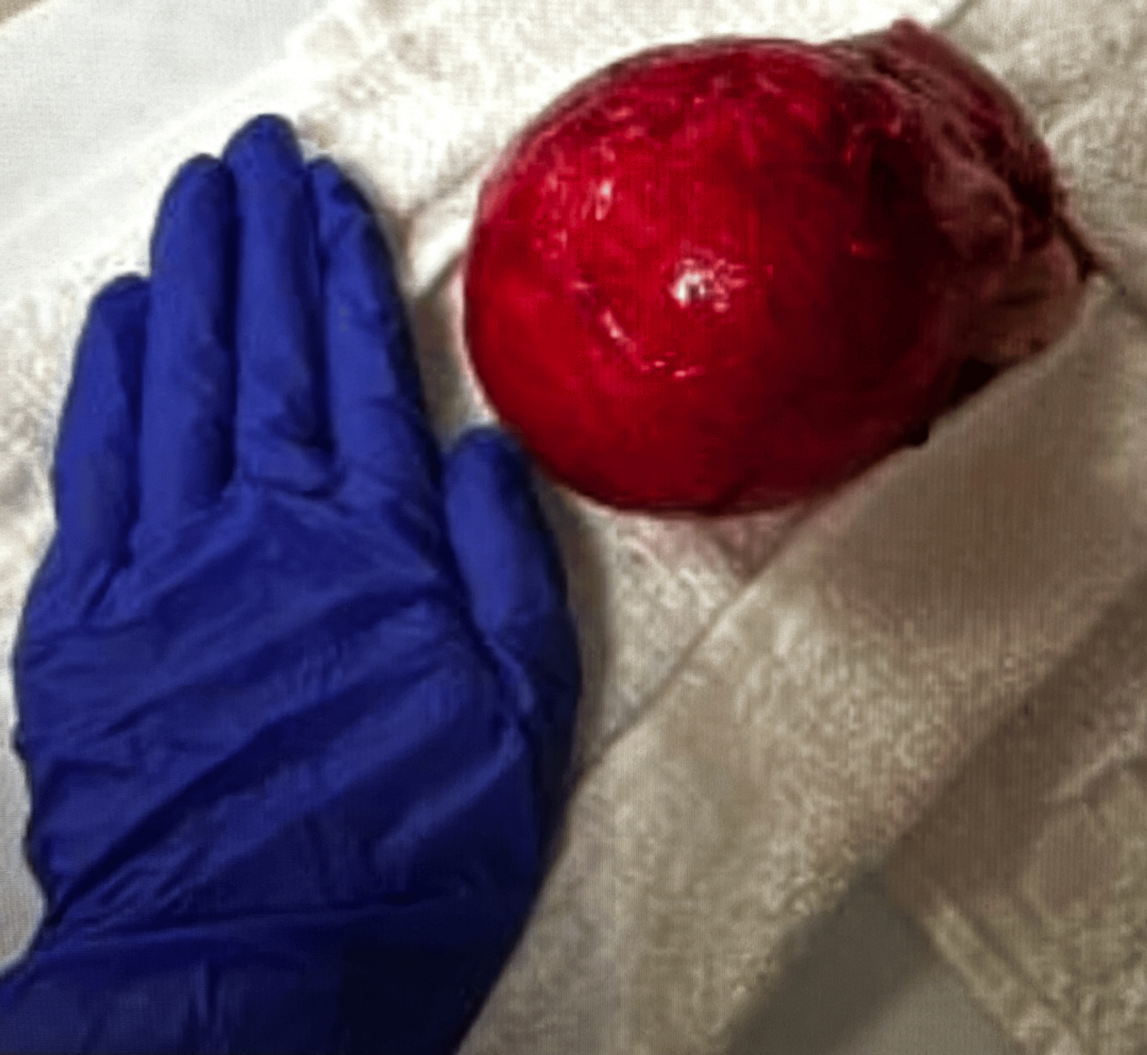
Large leiomyoma (9.8 cm x 8.9 cm x 8.4 cm) in the lower uterine section (paracervical) with a hand for size comparison

**Figure 3:**
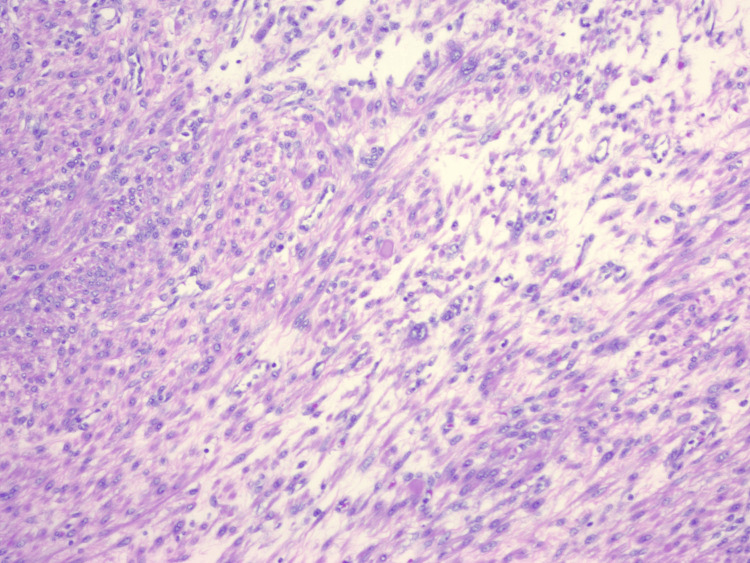
Uterine leiomyomas can show similar features to the renal cell tumors including prominent eosinophilic nucleoli with perinuclear halos

Pelvic ultrasonography demonstrated multiple small left-sided uterine leiomyomas (Figure 1) and a large posterior uterine mass (Figure 2). Based on imaging and clinical findings, the patient underwent a total hysterectomy with left salpingectomy. This route of surgery was made to minimize future risk to the patient after surgery. For presumed adenomyosis, the pathology report revealed acute and chronic cervicitis with squamous metaplasia, benign secretory endometrium, and an unremarkable left fallopian tube. Notably, pathologic evaluation identified a 9.8 cm leiomyoma with FH-deficient-like morphology (9.8 cm) (Figure 3). Additional benign leiomyomas up to 2.1 cm were identified. Sections of the largest leiomyoma demonstrated features suggestive of FH-deficient morphology, including alveolar edema, staghorn vessels, schwannoma light growth, patchy bizarre nuclei, and eosinophilic cytoplasmic inclusions. No increased mitotic activity or tumor necrosis was identified. These features may be associated with either a sporadic or a germline FH mutation. 

The gross description of the hysterectomy was 8.6 cm from fundus to ectocervix, 6.2 cm from cornu to cornu, and 4.3 cm from anterior to posterior with a trimmed weight of 496 g. Gross description of the largest leiomyoma revealed a large, well-circumscribed nodule involving the left paracervical, parametrial, and subserosal tissue of the uterus, measuring 9.8 x 8.9 x 8.4 cm, diffusely adherent and focally hemorrhagic. The irregular ovoid nodule was located within 0.3 cm of the supracervical surgical margin. The cut surface was yellow-tan to gray-white and glistening with a thin, clear mucoid film exuding upon sectioning. The nodule did not significantly displace the myometrium, but was tightly adherent to the parametrium within 2.6 cm of the left cornu. Additional well-circumscribed nodules were present in the anterior and posterior myometrium up to 2.1 cm. Their cut surfaces were gray-white, focally hyperemic, and whorled. A total of six intramural and subserosal nodules were identified. 

The endometrium is yellow-tan to brown, glistening, and 0.2 cm maximum thickness. No polypoid structure or lesion is readily visible. The ectocervical mucosa of the separately submitted cervical stump is gray-white to pink, smooth and glistening. The endocervical mucosa is tan-white without small cyst-like structures. There is no evidence of a granular mass or polypoid structure present in the small amount of endocervical canal at the transitional zone. The separate fallopian tube is 6.6 x 0.7 cm with unremarkable serosa, fimbria, and cut surfaces. There is no other fallopian tube or adnexal structure in the container. The fimbria will be bisected, and sample sections will be submitted of the stated left fallopian tube. Immunohistochemistry was not yet performed. A urology referral was not yet done, and so there was no imaging of the kidneys. The patient did not have RCC at time of presentation.

## Discussion

FH-deficient leiomyomas are a rare subset of leiomyomas and account for 0.4-1.6% of all cases [4]. Due to the absence of germline FH mutation testing, we cannot definitively state that the patient has HLRCC syndrome. However, the pathological slide analysis provides very strong evidence that our case is, in fact, consistent with HLRCC syndrome. We speculate that the characteristics of bizarre nuclei atypia and additional histopathologic features, including staghorn blood vessels, alveolar edema, and eosinophilic cytoplasmic inclusions, provide strong support for an FH-deficiency leiomyoma associated with HLRCC. We correlate that due to the extreme size and unusual anatomic location, there may have been an increased risk for subsequent development of HLRCC-associated RCC if no intervention, such as hysterectomy, had been performed. We hypothesize that by choosing to leave the excision site open after removal of the large paracervical leiomyoma, the patient may see an improved clinical prognosis in the future as the opening allows for more complete drainage.

We postulate that the unique location of this large leiomyoma, posterior to the lower uterine segment and with near-retroperitoneal extension and adherence to bladder and ureters, may give some insight into the patient presentation and pathophysiology of HLRCC; the histopathologic features of this FH-deficient leiomyoma mirrored findings reported in HLRCC-associated RCCs. In particular, the bizarre nuclei and eosinophilic cytoplasmic inclusions may support our hypothesis that FH-deficient leiomyomas contribute to the biologic milieu predisposing to the development of RCC [7]. We also speculate that the left paracervical location of the large leiomyoma may have caused a rightward uterine shift of the uterus, manifesting as sharp referred pain in the right lower quadrant of the patient through compression of the appendix [8]. We correlate that a rightward uterus shift may have also led to restriction of the right fallopian tube, producing a viscerosomatic reflex at T10-T11, corresponding to the same segmental level as the right kidney, and potentially contributing to the patient’s right flank pain [9]. The right lower quadrant pain may also have been a response from impingement on retroperitoneal structures, including the proximal colon, cecum, and appendix, all of which share viscerosomatic innervation at thoracic sympathetic levels of T10-T11, potentially accounting for the right flank pain [10]. 

Although from different embryologic origins, the smooth muscle layer of the uterus and the kidneys are both derived from mesoderm. When either tissue harbors germline or sporadic mutations in the FH gene, the result is FH-deficient cells demonstrating bizarre nuclei; both tissues may exhibit marked similarity, particularly bizarre nuclei atypia with eosinophilic cytoplasmic inclusions [11]. Could our specific case of this extraordinarily large FH-deficient leiomyoma in this unusual location help provide further insight into the pathophysiology underlying the potential progression toward HLRCC-associated RCC? We hypothesize that a large FH-deficient leiomyoma originated from or created a conduit between the reproductive and renal systems, thereby allowing the transfer of these FH-deficient cells between these compartments. 

The patient did not have any notable skin lesions consistent with HLRCC. Proper imaging was undertaken, which included an ultrasound. A CT/MRI was not obtained, as clinical history, examination, and ultrasound provided the OB-GYN with sufficient information to perform a total abdominal hysterectomy with left salpingectomy. Although Immunohistochemistry was not completed, the pathology report clearly demonstrates findings consistent with HLRCC syndrome and FH-deficient leiomyoma. The pathologist’s comments indicate a comparison of our case with similar reports. The patient was advised to follow up with genetic counseling to further evaluate HLRCC syndrome. The patient was educated regarding the importance of continued monitoring and surveillance per guidelines regarding monitoring for HLRCC syndrome.

## Conclusions

We present a unique case of an extremely low-lying and large uterine leiomyoma with FH-deficient morphology located posteriorly and paracervically, measuring 9.8 cm, which exceeds the uterine longitudinal length of 8.6 cm. Based on a detailed clinical examination, ultrasound, and the experience of the OB-GYN, the diagnosis of a leiomyoma was made. The pathological value of the case is in the discovery of the leiomyoma showing FH-deficient morphology. The clinical value of the case is the unique paracervical location of the large FH-deficient leiomyoma. These diagnostic, pathological, and clinical findings have contributed to our hypothesis regarding the pathophysiology of disease progression in HLRCC syndrome. A limitation is that germline FH mutation testing was not performed, so the definitive diagnosis of HLRCC remains unconfirmed. 

The unique clinical finding in this case was that the leiomyoma with FH-deficient morphology compressed adjacent structures, including the bladder and ureters, and demonstrated near retroperitoneal extension. In addition, it is known that FH-deficient leiomyomas can potentially metastasize. We hypothesize that interactions with the renal system may contribute to the development of this FH-deficient leiomyoma, given its close anatomic proximity and shared histopathologic features with renal tissue. HLRCC-associated RCCs exhibit many of the same pathological features as FH-deficient leiomyomas, including bizarre nuclei, staghorn vessels, and eosinophilic cytoplasmic inclusions. Finally, we hypothesize that the clinical decision to leave the excision site open after complete excision of the leiomyoma may provide a better clinical prognosis for the patient by allowing for drainage.
